# GeneralizedDTA: combining pre-training and multi-task learning to predict drug-target binding affinity for unknown drug discovery

**DOI:** 10.1186/s12859-022-04905-6

**Published:** 2022-09-07

**Authors:** Shaofu Lin, Chengyu Shi, Jianhui Chen

**Affiliations:** 1 Faculty of Information Technology, Beijing University of Technology, No. 100, Pingleyuan, Chaoyang District, Beijing, 100124 China; 2 Beijing International Collaboration Base on Brain Informatics and Wisdom Services, Beijing University of Technology, No. 100, Pingleyuan, Chaoyang District, Beijing, 100124 China; 3 Beijing Key Laboratory of MRI and Brain Informatics, Beijing University Of Technology, No. 100, Pingleyuan, Chaoyang District, Beijing, 100124 China

**Keywords:** DTA prediction, Pre-training task, Multi-task learning, Dual adaptation mechanism

## Abstract

**Background:**

Accurately predicting drug-target binding affinity (DTA) in silico plays an important role in drug discovery. Most of the computational methods developed for predicting DTA use machine learning models, especially deep neural networks, and depend on large-scale labelled data. However, it is difficult to learn enough feature representation from tens of millions of compounds and hundreds of thousands of proteins only based on relatively limited labelled drug-target data. There are a large number of unknown drugs, which never appear in the labelled drug-target data. This is a kind of out-of-distribution problems in bio-medicine. Some recent studies adopted self-supervised pre-training tasks to learn structural information of amino acid sequences for enhancing the feature representation of proteins. However, the task gap between pre-training and DTA prediction brings the catastrophic forgetting problem, which hinders the full application of feature representation in DTA prediction and seriously affects the generalization capability of models for unknown drug discovery.

**Results:**

To address these problems, we propose the GeneralizedDTA, which is a new DTA prediction model oriented to unknown drug discovery, by combining pre-training and multi-task learning. We introduce self-supervised protein and drug pre-training tasks to learn richer structural information from amino acid sequences of proteins and molecular graphs of drug compounds, in order to alleviate the problem of high variance caused by encoding based on deep neural networks and accelerate the convergence of prediction model on small-scale labelled data. We also develop a multi-task learning framework with a dual adaptation mechanism to narrow the task gap between pre-training and prediction for preventing overfitting and improving the generalization capability of DTA prediction model on unknown drug discovery. To validate the effectiveness of our model, we construct an unknown drug data set to simulate the scenario of unknown drug discovery. Compared with existing DTA prediction models, the experimental results show that our model has the higher generalization capability in the DTA prediction of unknown drugs.

**Conclusions:**

The advantages of our model are mainly attributed to two kinds of pre-training tasks and the multi-task learning framework, which can learn richer structural information of proteins and drugs from large-scale unlabeled data, and then effectively integrate it into the downstream prediction task for obtaining a high-quality DTA prediction in unknown drug discovery.

## Background

Drug discovery is very inefficient by traditional wet laboratory experiments [1, 2]. It usually spends 10–17 years and billions of dollars on research and experimental processes [3]. Such an inefficient process is obviously difficult to meet the needs of rapidly developing diseases, such as COVID-19. In order to improve the efficiency of drug discovery, predicting drug-target interaction (DTI) in silico has attracted more and more attention [2, 4–7]. These computational DTI prediction methods not only have low cost but also can greatly accelerate the process of drug development [8].

Predicting drug-target binding affinity (DTA) [9] is a kind of special DTI prediction task. Unlike traditional DTI prediction based on binary classification, DTA prediction can obtain the quantitative binding affinity between drugs and targets, which provides more detailed descriptions about drug-target interactions. Related studies mainly adopted machine learning models to realize a two-stage modeling process, including encoding and decoding. The encoding process learns feature representations from drugs and various targets, such as proteins. The decoding process predicts the binding affinity based on these feature representations. Early studies often adopted shallow machine learning models to learn feature representations for DTA prediction. SimBoost [10] calculated the affinity similarity between drug compounds and targets by using collaborative filtering and then used the similarity as the feature vector to predict DTA. KronRLS [11] used kernel-based methods to generate molecular descriptors of drugs. With the rapid development of deep learning, the deep neural networks have been widely used in DTA prediction, especially in the encoding process. DeepDTA [12] introduced deep learning into DTA prediction for the first time, which used convolutional neural network (CNN) to generate 1D representations of drugs and proteins. GraphDTA [13] used the open source chemical informatics software RDKit to construct the molecular graph of drug compounds instead of the compound string, and learnt the feature vector of drug compounds by using graph neural network. MGraphDTA [14] built a super-deep GNN with 27 graph convolutional layers to capture the local and global structure of the compound simultaneously. MATT$\_$DTI [15] encoded the correlations between atoms of drug compounds by a relation-aware self-attention block and modeled the interaction of drug representations and target representations by the multi-head attention block. DeepNC [16] learnt the features of drugs and targets by the layers of GNN and 1-D convolution network, respectively. MINN-DTI [17] combined an interacting-transformer module with an improved Communicative Message Passing Neural Network (CMPNN) to better capture the two-way impact between drugs and targets. Besides feature coding of drugs and proteins, feature aggregation has also attracted attention. FusionDTA [18] utilized a novel muti-head linear attention mechanism to aggregates global information based on attention weights.

All of the above studies are based on labelled drug-target data sets, such as Davis [19] and Kiba [20]. Compared with tens of millions of compounds and hundreds of thousands of proteins, labelled drug-target data are relatively limited. The Davis data set [19] only contains 72 drugs and 442 targets. The KEGG data set [21] only has a total of 4797 drug-target pairs. However, the ZINC15 database [22] contains over 230 million compounds in ready-to-dock. It is difficult to learn feature representations covering all drugs and compounds only based on relatively small labelled drug-target data. Aiming at this problem, Hu et al. [23] performed the protein pre-training task on large amounts of unlabelled data to obtain the robust protein encoding model with enhanced structural information of amino acid sequences, and then fine-tuned the encoding model on the decoding process, i.e., the DTA prediction modeling process, for fitting the relatively small labelled drug-target data. Owing to enhanced structural information, their DTA prediction model achieved excellent results.

However, Hu et al.’s model only obtained structural information about amino acid sequences by using the protein pre-training task and neglected structural information of molecular graphs of drug compounds. More importantly, there is a task gap between pre-training and DTA prediction. The goal of protein pre-training is to accurately predict masked amino acids based on context information of amino acid sequences, but the goal of DTA prediction is to accurately calculate the binding affinity between drug compounds and proteins. Hu et al. adopted a sequential structure to integrate the pre-training task and the DTA prediction task [23]. The task gap between them can bring the catastrophic forgetting problem [24]. As the number of fine-tuning iterations increases, the downstream prediction model increasingly focuses on the drugs and proteins appearing frequently in the labelled drug-target training data, resulting in poor prediction results on those unknown drugs, which never appear in the labelled drug-target data. This is a kind of out-of-distribution (OOD) problems in biomedicine [25]. The DTA prediction model has the poor generalization capability [26] on unknown drug discovery. This problem is particularly serious when labelled data are obviously smaller than unlabeled pre-training data.

However, existing studies on DTI and DTA prediction did not pay special attention to these unknown drugs. To our knowledge, the poor generalization ability of model in unknown drug discovery has not been studies. In order to prove the existence of this problem, we used the Davis data set to perform a DTA prediction task for unknown drug discovery. The original training and test sets were divided referring to Öztürk et al.’s work [13]. We randomly selected 20$\%$ of drugs in the original training set, a total of 14 kinds of drugs, as new drugs. All corresponding drug-target pairs were deleted from the original training set to construct an unknown drug training set. The corresponding 5178 drug-target pairs were extracted from the original test set to construct an unknown drug test set. The DTA prediction task in unknown drug discovery was performed on the unknown drug training and test sets. Using GraphDTA [12] to iterate 1000 times, the results are as follows.

**Fig. 1:**
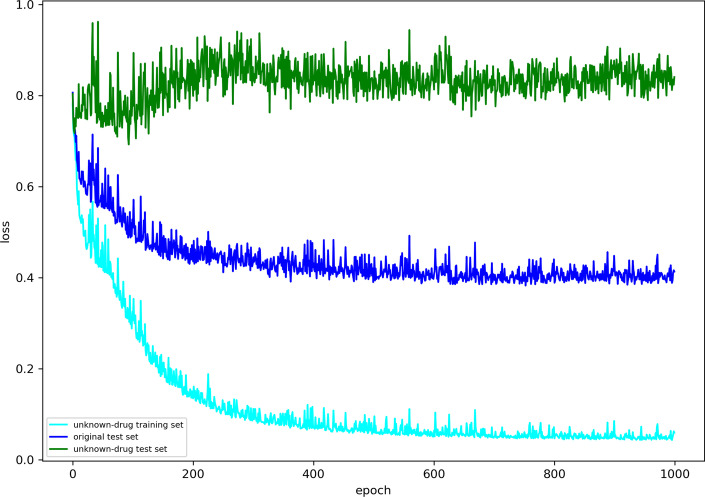
Convergence analysis of GraphDTA in unknown drug discovery

Figure 1 shows the convergence curve of loss function in 1000 times of iterations. The horizontal axis represents the number of iterations and the vertical axis represents the value of loss function. As shown in this figure, the losses on the unknown drug training set and the original test set decrease significantly in the first 200 iterations, the loss on the unknown drug test set fluctuates repeatedly at 0.85 and has no downward trend. This indicates that GraphDTA is over fitted and lacks the sufficient generalization capability for unknown drug discovery. It is necessary to carry out special studies on this problem.

In previous studies, overfitting of model can be intervened by means of data enhancement, feature removal, and so on. For the DTA prediction task, data enhancement is too expensive because it needs to increase labelled drug-target data. Feature removal may reduce the accuracy of model and deviates from the original intention of feature enhancement of pre-training. Based on the above observations, this study proposes a new DTA prediction model, called GeneralizedDTA, by combining self-supervised pre-training and multi-task learning. The main contributions can be summarized as follows:

1. Firstly, this study introduces both protein and drug pre-training tasks into the DTA prediction task. By using these two kinds of pre-training tasks, structural information of both amino acid sequences of proteins and molecular graphs of drug compounds is learnt and integrated in the DTA prediction task for the first time.
2. Secondly, this study develops a multi-task learning model with a dual adaptation mechanism for alleviating the catastrophic forgetting problem of pre-training parameters. By using the MAML-based updating strategy, pre-training parameters are adapted by a few gradient updates, and then with the updated parameters, the whole model is trained in the downstream DTA prediction task for accelerating convergence and preventing the model from falling into local optimality.
3. Thirdly, this study constructs a group of unknown drug data sets to simulate a scenario of unknown drug discovery and performs comparative experiments on these data sets. The experimental results show that the generalization capability of our model has been significantly improved compared with existing DTA prediction models. It can be better adapted to DTA prediction in unknown drug discovery.

## Methods

**Fig. 2:**
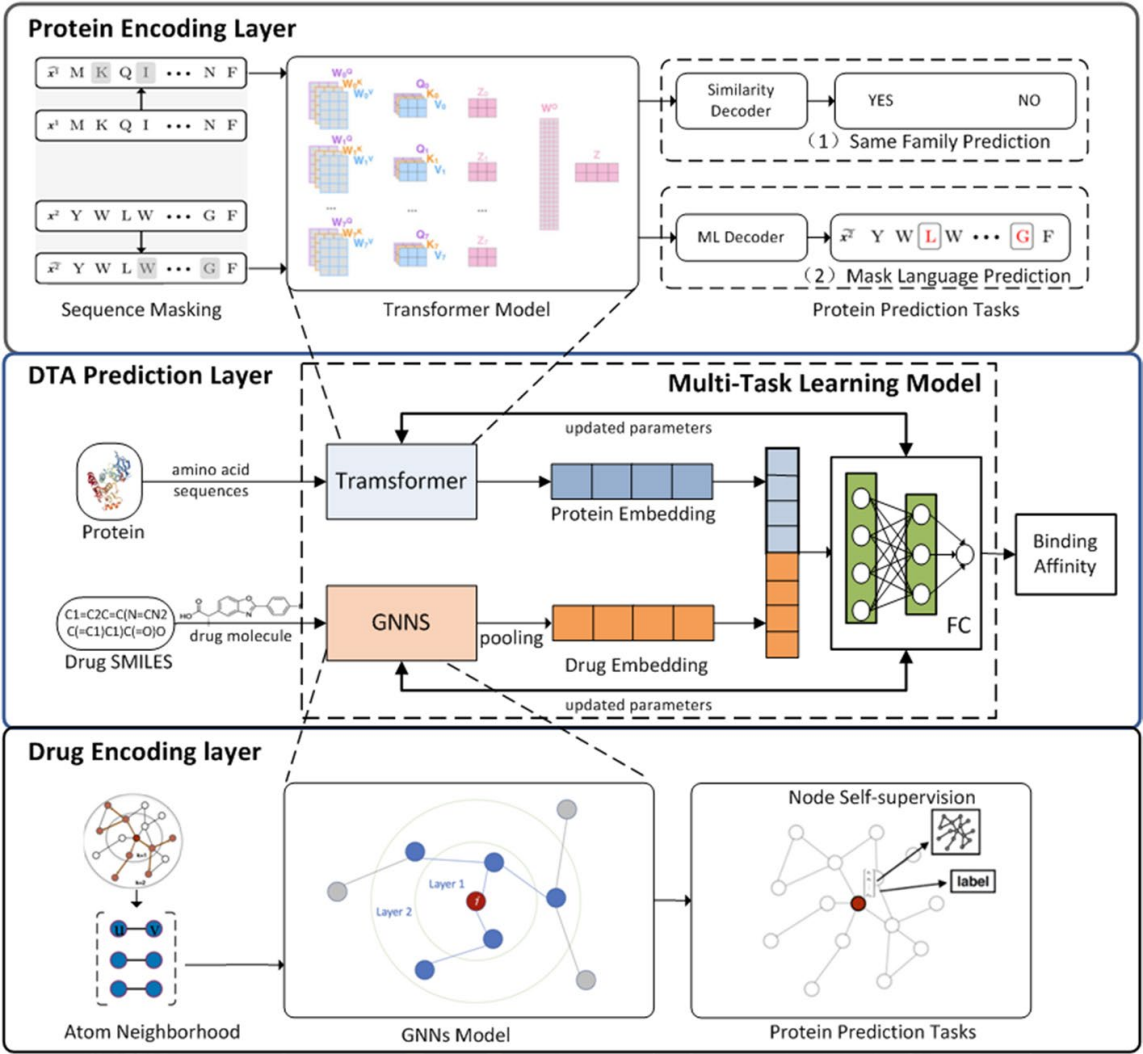
The model architecture of GeneralizedDTA

In order to realize DTA prediction in unknown drug discovery, this study proposes the GeneralizedDTA model by combining self-supervised pre-training and multi-task learning. Two kinds of protein pre-training tasks are adopted to learn structural information of amino acid sequences. A kind of new drug pre-training task is designed to learn structural information of molecular graphs of drug compounds. In order to alleviate the catastrophic forgetting problem of pre-training parameters, a multi-task learning framework with a dual adaptation mechanism is developed to prevent the prediction model from falling into overfitting. Figure 2 gives the model architecture of GeneralizedDTA, which includes four modules: the protein encoding layer, the drug encoding layer, the DTA prediction layer, and the multi-task learning framework.

### Protein encoding layer

The protein encoding layer encodes amino acid sequences of proteins as vectors by using protein pre-training tasks. Inspired by BERT [27], this study adopts a transformer model with the multi-head attention as the encoder to receive amino acid sequences. Given a amino acid sequence $t=\left[ t_{1}, \ldots , t_{n}\right]$ where $t_{i} \in \{21$ amino acid types$\}$, the transformer model converts it into $z=\left[ z_{1}, \ldots , z_{n}\right]$ as follows:

1 $$\begin{aligned}&z={\text {Transformer}}(Q, K, V ; t)=\text {Concat}\left( \text {head}_{1}, \ldots , \text {head}_{n}\right) W^{\circ } \end{aligned}$$

2 $$\begin{aligned}&\text{ head}_{i}=\text{ Attention }(Q, K, V) \end{aligned}$$

3 $$\begin{aligned}&{\text {Attention}}(Q, K, V)={\text {softmax}}\left( \frac{Q K^{T}}{\sqrt{d_{k}}}\right) V \end{aligned}$$

where $Q \in R^{d_{1} \times d_{2}}, K \in R^{d_{1} \times d_{2}}, V \in R^{d_{1} \times d_{2}}$ are the parameters of attention, *n* is the number of heads, $W^{o} \in R^{d_{1}} \times d_{1}$ is the weight of heads and $\sqrt{d_{k}}$ is the dimension number of Q. The self-attention function is computed on the dot products of each queries with all keys simultaneously, and divided by a softmax function to obtain the weights on the values [28]. It can be simplified as a parameterized function Transformer ($\bullet$) with the parameter set $\theta$ :

4 $$\begin{aligned} z=\text{ Transformer }(\theta ; t), \theta =\left\{ Q, K, V, W^{o}\right\} \end{aligned}$$

Based on the transformer model, this study adopts two pre-training tasks to obtain structural information of amino acid sequences of proteins.

Masked Language Modeling (MLM) Task [28]: this task screens some amino acids at random and predicts their types. Given a masked amino acid sequence *t* and a masked amino acid set $m=\left\{ m_{1}, m_{2}, \ldots , m_{N}\right\}$, the MLM decoder calculates the log probability for *t* as follows:

5 $$\begin{aligned} z= & {} \text{ Transformer }(\theta ; t) \end{aligned}$$

6 $$\begin{aligned} m^{\prime }= & {} F C\left( \theta _{1}; z\right) \end{aligned}$$

where $F C(\bullet )$ is a fully connected neural network (FC) with the parameter $\theta _{1}$ and $m^{\prime }=\left\{ m_{1}^{\prime }, m_{2}^{\prime }, \ldots , m_{N}^{\prime }\right\}$ represents the predicted amino acid set for the whole masked amino acid set. Then the log-likelihood function is used as the evaluation metrics for the MLM task:

7 $$\begin{aligned} {\mathcal {L}}^{\mathbf {M L M}}\left( \theta , \theta _{1} ; m\right) =-\left[ \sum _{i=1}^{N} m_{i}^{\prime } \ln m_{i}+\left( 1-m_{i}^{\prime }\right) \ln \left( 1-m_{i}\right) \right] \end{aligned}$$

By the above MLM task, the transformer model could effectively learn the bidirectional contextual representation of amino acid sequences of proteins.

Same Family Prediction (SFP) Task [29, 30]: this task enables the model to determine if two proteins belong to the same family. In order to pre-train the transformer model with the SFP task, this study selects two amino acid sequences $t^{1}$ and $t^{2}$ from the Pfam dataset. Random sampling is adopted to ensure the probabilities that they come from the same class and different classes are the same. Aiming at the protein pair $\left\langle \mathrm {t}^{1}, \mathrm {t}^{2}\right\rangle$, a FC with dropout [31] is used to calculate their similarity value:

8 $$\begin{aligned} {\hat{c}}=F C\left( \theta _{2} ; z_p\right) \end{aligned}$$

where $\theta _{2} \in {\mathbb {R}}^{|z| \times 2}$ is the parameter of FC, $z_p=\left[ z_{1}^{1}, \cdots , z_{1_{1}}^{1}, z_{1}^{2}, \cdots , z_{n_{2}}^{2}\right] z \in {\mathbb {R}}^{|z|\times 1}$ is the vector representation of $\left\langle \mathrm {t}^{1}, \mathrm {t}^{2}\right\rangle$ and ${\hat{c}} \in {\mathbb {R}}^{2 \times 1}$ is the predicted similarity value, i.e., a probability that the protein pair belongs to the same protein family. The SFP task trains the model to minimize the cross-entropy loss which is designed to deal with predicted errors on probabilities. Therefore, this study adopts the log-likelihood function to measure the SFP loss:

9 $$\begin{aligned} {\mathcal {L}}^{\mathrm {SFP}}\left( \theta , \theta _{2} ; t\right) =-\ln p\left( n=n_{i} \mid \theta , \theta _{2}\right) , \quad n_{i} \in [ \text{ same } \text{ family, } \text{ not } \text{ same } \text{ family}] \end{aligned}$$

As the transformer model is asked to produce the higher similarity value for proteins from the same family, the SFP task enables the transformer model to better absorb global structural information of amino acid sequences of proteins.

### Drug encoding layer

The drug encoding layer encodes molecular graphs of drug compounds as vectors by a brand-new drug pre-training task. It adopts GCN [32] to mine potential relationships from molecular graphs of drug compounds.

Given a molecular graph of drug compound ${\mathcal {G}}=({\mathcal {V}}, {\mathcal {E}}, {\mathcal {X}}, {\mathcal {Z}})$ where ${\mathcal {V}}$ is the chemical atom set, ${\mathcal {E}}$ is the chemical bond set, ${\mathcal {X}} \in {\mathbb {R}}^{|\nu | \times d_{v}}$ and ${\mathcal {Z}} \in {\mathbb {R}}^{|\varepsilon | \times d_{e}}$ are the atom and bond feature sets, respectively. GCN is mainly involved with two key computations “update” and “aggregate” for each atom at every layer. They can be represented as a parameterized function $\Psi (\bullet )$ with the parameter $\psi$ :

10 $$\begin{aligned} \begin{aligned} {\mathbf {h}}_{v}^{l}&=\Psi (\psi ; {\mathcal {A}}, {\mathcal {X}}, {\mathcal {Z}})^{l}\\&={\text {UPDATE}}\left( {\mathbf {h}}_{v}^{l-1}, {\text {AGGREGATE}}\left( \left\{ \left( {\mathbf {h}}_{v}^{l-1}, {\mathbf {h}}_{w}^{l-1}, {\mathbf {z}}_{w v}\right) : u \in {\mathcal {N}}_{v}\right\} \right) \right) \end{aligned} \end{aligned}$$

where $u, v \in {\mathcal {V}}$ are two chemical atoms, $z_{u v}$ is the feature vector of the chemical bond (*u*, *v*), $\mathrm {h}_{v}^{0}=\mathrm {x}_{v} \in {\mathcal {X}}$ is the input of GCN and represents the feature of atom *v*, $\mathrm {h}_{v}^{l}$ represents the feature of atom *v* on the l-th layer of GCN, ${\mathcal {A}}$ is the adjacency matrix of drug compound ${\mathcal {G}}$, and ${\mathcal {N}}_{v}$ is the neighborhood atom set of atom *v*.

In order to get a representation of drug compound ${\mathcal {G}}$, the POOLING function on the last GCN layer is used to transform the molecular graph into a vector:

11 $$\begin{aligned} {\mathbf {h}}_{{\mathcal {G}}}={\text {POOLING}}\left( \left\{ {\mathbf {h}}_{v}^{l} \mid v \in {\mathcal {V}}\right\} \right) \end{aligned}$$

where $h_{{\mathcal {G}}}$ is the vector representation of drug compound ${\mathcal {G}}$ , POOLING is a simple pooling function like the max or mean-pooling [33, 34]. For simplicity, we represent GCN as follows:

12 $$\begin{aligned} {\mathbf {h}}_{{\mathcal {G}}}=G C N (\psi ; {\mathcal {G}}) \end{aligned}$$

Based on the GCN model, this study designs a new pre-training task to learn structural information of molecular graphs of drug compounds.

**Fig. 3:**
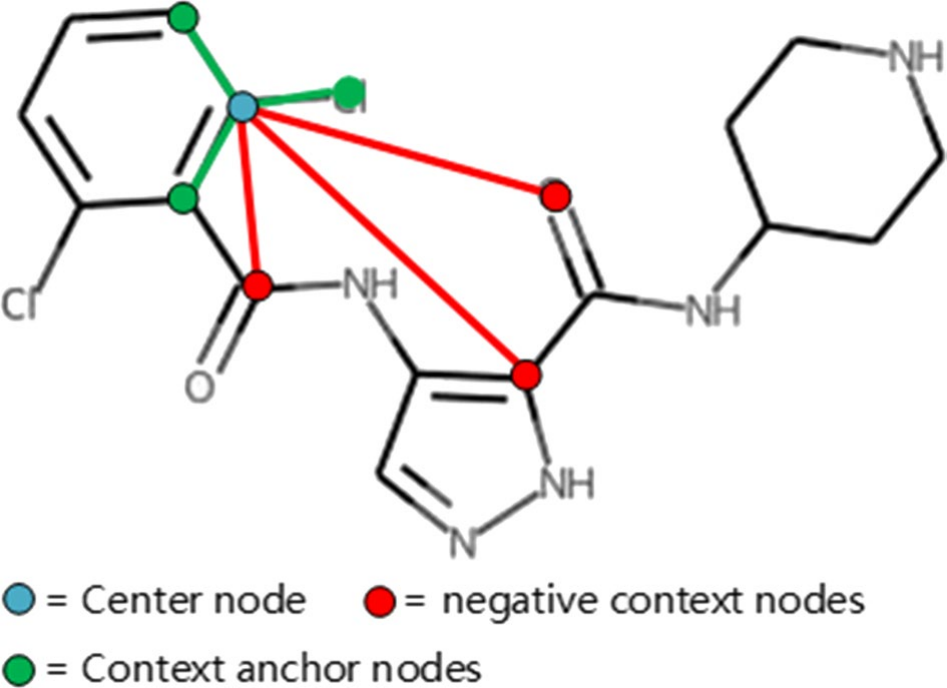
The drug pre-training based on context prediction

Drug Pre-training (DP) Task: this new task is designed to improve the representation learning capability on drugs by encouraging the generation of similar embeddings for neighboring chemical atoms in the molecular graph of drug compounds [35]. The aggregation is a key computation in each layer of GCN. In compound-level aggregation, the neighboring chemical atoms aggregate their information based on Eq. (10) [36, 37]. For each chemical atom $v \in {\mathcal {V}}$, GCN gets its representation by $\mathrm {h}_{v}$ and $\Psi (\bullet )$ in Eq. (10). Therefore, as shown in Fig.3, given a random atom bond *u* as the center node, the self-supervised loss function [38] is chosen to realize the DP task, i.e., encourage similar embeddings for neighboring chemical atoms:

13 $$\begin{aligned} {\mathcal {L}}^{\text{ atom } }\left( \psi ; \mathcal {{\mathcal {G}}}\right) =\sum _{(u, v) \in {\mathcal {G}}}-\ln \left( \sigma \left( {\mathbf {h}}_{u}^{\top } {\mathbf {h}}_{v}\right) \right) -\ln \left( \sigma \left( -{\mathbf {h}}_{u}^{\top } {\mathbf {h}}_{v^{\prime }}\right) \right) \end{aligned}$$

where *v* is the context anchor node which is directly connected to the center node *u*, $v^{\prime }$ is the negative context node which is not directly connected to *u*, $\psi$ is the parameter of GCN, and $\sigma$ is the sigmoid function. By 5 layers of GCN, each atom embedding absorbs almost all small local structures in the molecular graph [39, 40].

### DTA prediction layer

The DTA prediction layer is to associate the drug compound with the protein for predicting their binding affinity. This study adopts a FC for DTA prediction. For a given drug-protein pair $\left\langle {\mathcal {G}}, t\right\rangle$ where ${\mathcal {G}}$ is a molecular graph of drug compound and *t* is an amino acid sequence, the corresponding drug compound vector ${\varvec{h}}_{\mathcal {G}}$ and the protein vector $z_p$ can be obtained by the drug encoding layer and the protein encoding layer. Then, the process of predicting their binding affinity ${\hat{y}}$ is shown as follows:

14 $$\begin{aligned} {\hat{y}}=F C\left( \gamma ; {\text {Concat}}\left( {\varvec{h}}_{\mathcal {G}}, z_p\right) \right) \end{aligned}$$

where $\gamma$ is the parameter of full connection layers and Concat ($\bullet$) indicates that the input is the concatenated vector of ${\varvec{h}}_{G}$ and $z_p$.

The DTA prediction task trains the model to minimize the loss function. This study adopts the mean squared error (MSE) as the loss function:

15 $$\begin{aligned} {\mathcal {L}}^{\text{ affinities } }(\theta , \psi , \gamma ;\left\langle {\mathcal {G}}, t\right\rangle )=\frac{1}{2}({\hat{y}}-y)^{2} \end{aligned}$$

where ${\hat{y}}$ is the predicted binding affinity of drug-protein pair and *y* is the true value, $\theta , \psi , \gamma$ are combined as model parameters.

### Multi-task learning framework with a dual adaptation mechanism

This study adopts multi-task learning to link the encoder, i.e. the pre-training tasks and the decoder, i.e. the DTA prediction task, for preventing overfitting caused by the local optimality under a relatively small supervised samples. In order to make the overall model bias against the main task DTA prediction, this study adopts the updated strategy of MAML [41].

The drug pre-training task is defined as the query set. For this task, we adjust the prior parameter $\psi$ of compound-level aggregation with one or a few gradient descent steps. The learning rate is set to $\alpha$ for dual adaptation. The new prior parameter $\psi ^{\prime }$ can be obtained as follows:

16 $$\begin{aligned} \psi ^{\prime }=\psi -\alpha \frac{\partial {\mathcal {L}}^{\text{ atom } }\left( \psi ; {\mathcal {G}}\right) }{\partial \psi } \end{aligned}$$

Then, the FC parameter $\gamma$ in the DTA prediction layer, which is defined as the support set, will be updated as follows:

17 $$\begin{aligned} \gamma ^{\prime }=\gamma -\alpha \frac{\partial {\mathcal {L}}^{\text{ affinities } }\left( \psi ^{\prime }, \gamma ;( {\mathcal {G}}, t)\right) }{\partial \gamma } \end{aligned}$$

After that, all the parameters are updated through the backpropagation of the overall loss function of the multi-tasking learning. We define the overall loss function as follows:

18 $$\begin{aligned} {\mathcal {L}}^{\text{ all } }=\lambda _{\text{ atom } } {\mathcal {L}}^{\text{ atom } }+{\mathcal {L}}^{\text{ affinities } } \end{aligned}$$

where $\lambda _{\text{ atom }}$ set manually is the weight of the loss function of drug pre-training task. This study updates all learnable parameters by gradient descent. Before the pre-training drug task, we record the original model parameters, and take the parameters (query set) updated for the first time in pre-training as the prior parameters of the subsequent DTA prediction. The comprehensive loss function of DTA prediction and the drug pre-training task is taken as the objective function of dual adaptation. Subsequent original parameters are updated through the multi-task learning framework. Different from the frozen-strategy, the updated model parameters are original parameters rather than prior parameters.

The dual adaptation mechanism needs to save all learnable parameters in the pre-training task. For multi-head transformers learning, this will bring a huge increase in training time. Furthermore, this study mainly focuses on unknown drugs and introduces the drug pre-training task into the DTA prediction. Therefore, the multi-task learning framework in this study only combines the drug pre-training task with the DTA prediction task by using the above dual adaptation mechanism.

## Results

### Data preparation

This study performed the pre-training tasks on the following two datasets:

- Protein pre-training dataset: The Pfam dataset [42] was used for protein pre- training. It was produced at the European Bioinformatics Institute using a sequence database, which is based on UniProt. Over 21M amino acid sequences of proteins were clustered into 16,479 families based on the sequence similar- ity. This study performed two protein pre-training tasks on this dataset for learning structural information of amino acid sequences.
- Drug pre-training dataset: The ZINC15 database [22] was used for drug pre-training. It is provided by the Irwin and Shoichet Laboratories at the University of California. In this study, 2 million unlabeled compounds was used for learning structural information of molecular graphs of drug compounds by the drug pre-training task.

The Davis [19] and Kiba [20] were selected for performance evaluation. The Davis dataset includes 30056 drug-target pairs and is involved with 442 proteins and 68 compounds. The Kiba dataset includes 118254 drug-target pairs and is involved with 229 proteins and 2068 compounds. Their binding affinities are indicated by the relevant inhibitors with their respective dissociation constant values. A group of unknown drug data sets were constructed for simulating the scenario of unknown drug discovery. The process includes the following two steps:

- Unknown drug compound/protein selection: This study selected unknown drug compounds and proteins based on the similarity. Referring to [43], we performed the substructural features based k-means algorithm on all the drug compounds and selected outliers as unknown drug compounds. Referring to [25], we selected unknown proteins based on the Pfam family. The proteins from the smallest 42 families were selected as unknown proteins.
- Unknown dataset construction: Those drug-target pairs containing any unknown compounds or any unknown protein were extracted as the unknown test set (unknown-TeS). The corresponding drug-target pairs were removed from original training set [12] and the remaining data were used to construct the unknown training set (unknown-TrS).

The distribution of data is shown in Table 1. Similarly, we can obtain the unknown drug data sets from Kiba, as shown in Table 2. After constructing the unknown drug data sets, we removed all unknown drug compounds from the drug pre-training dataset to avoid data leakage.

**Table 1:** The data distribution in the unknown drug data sets from Davis

	Number of proteins	Number of drugs	Number of drug-target pairs
All data	442	68	30056
unknown-TrS	369	56	20664
unknown-TeS	442	68	10409

**Table 2:** The data distribution in the unknown drug data sets from Kiba

	Number of proteins	Number of drugs	Number of drug-target pairs
All data	229	2068	118254
unknown-TrS	191	1723	82524
unknown-TeS	229	2068	32490

### Model parameters

Protein pre-training tasks were first performed alone, and then the drug pre-training task and the DTA prediction task were carried out at the same time, by using the multi-task learning framework and dual adaptation mechanism. For protein encoding, the dimension size of amino acid vector was set to 20, the number of self-attention heads was 12, the number of hidden layers was 12, and the dimension size of hidden layer was 768. For drug encoding, the lay number of GCN was set to 5, and the dimension size of hidden layer was 300. For DTA prediction, the layer number of FC was set to 3. For multi-task learning, the learning rate was set to 0.001 and the weight of drug pre-training was set to 0, 0.5, 1.0 and 2.0, respectively.

### Baseline methods

In order to prove the validity of model, this study compares the proposed GeneralizedDTA with the following baseline methods:

- DeepDTA [12]: It used CNN and the pooling architecture to capture the potential interaction features between proteins and drugs. Research showed that the CNN network with a smaller number of parameters can be used to test overfitting of transformer. Therefore, this study adopted three layers of convolution for drug and protein encoding of DeepDTA, and the kernel sizes were set to 4,6,8, respectively.
- GraphDTA [13]: It represented SMILES strings of drugs as short ASCII strings. In this study, drug encoding of GraphDTA adopted three layers of graph convolution and the numbers of feature dimensions of layers were set to 78,156,312, respectively. This kind of incremental parameter design can enhance the information transfer between atoms.
- SAGDTA [44]: It exploited the self-attention mechanism on drug molecular graphs to obtain efficient representations of drugs. In this study, features of each atom node in the molecular graph and the SAG used the hierarchical pooing architecture with 3 blocks which has been demonstrated to absorb global information better.
- MGraphDTA [14]: It adopted a deep multiscale graph neural network based on chemical intuition for DTA prediction. A super-deep GNN with 27 graph convolutional layers was built to capture the local and global structure of the compound simultaneously. In this study, learning ration and embedding size were set to 5e-4 and 128 respectively.

### Evaluation metrics

This study adopted MSE and R-squared ($R^{2}$) [45] to evaluate the prediction results of the model. MSE and $R^{2}$ are well-defined metrics to measure how close the fitted line is in the regression task. They can be calculated as follows:

19 $$\begin{aligned} \mathrm {MSE}= & {} \frac{1}{n} \sum _{i=1}^{n}\left( y_{i}-{\hat{y}}_{i}\right) ^{2} \end{aligned}$$

20 $$\begin{aligned} R^{2}= & {} 1-\frac{\sum _{i}\left( y_{i}-{\hat{y}}_{i}\right) ^{2}}{\sum _{i}\left( y_{i}-{\bar{y}}\right) ^{2}} \end{aligned}$$

where ${\hat{y}}_{i}$ is the true value of binding affinity of the *i*-th drug-target pair, ${\hat{y}}$ is the corresponding predicted value, and ${\bar{y}}$ is the average of true values of all binding affinities.

### Performance evaluation on predicting drug-target binding affinity

Tables 3 and 4 give experimental results. It can be seen that SAGNet and our model with $\lambda _{\text{ atom } }=0$ have the worst performance in two datasets. It indicates that deeper networks without additional auxiliary constraints perform worse on unknown data. Our model with $\lambda _{\text{ atom } }=0$, in which $\lambda _{\text{ atom } }=0$ means the unbinding between drug pre-training and DTA prediction, had the biggest MSE. This indicates that overfitting exists due to catastrophic forgetting between drug pre-training and DTA prediction. It is necessary to develop a multi-task learning framework for binding pre-training and prediction models. GraphDTA [13] achieved the better performance than DeepDTA, indicating that structural information based on the molecular graph of drug compounds are valuable for DTA prediction. MGraphDTA [14] achieved the best results in four baseline methods. This proves once again the importance of structure information of the compounds, which is the important motivation to introduce the graph-based drug pre-training task in this study. Our model with $\lambda _{\text{ atom } }=0.5$ and $\lambda _{\text{ atom } }=1.0$ achieved the best performance in terms of all evaluation metrics in the Davis dataset and the Kiba dataset respectively. This shows that our model, which adopts a new drug pre-training task and combines it with the DTA prediction task by a multi-task learning framework, has better generalization capability in unknown drug discovery. But, different optimization weights may be required for different data sets. The reason can be attributed to the different affinity measurement methods in different datasets.

**Table 3:** Experimental results in the unknown drug data sets from Davis

Model	MSE	$R^{2}$
DeepDTA	1.0271	0.1454
GraphDTA	0.8872	0.2037
SAGDTA	1.1324	0.1654
MGraphDTA	0.8532	0.2287
Our method ($\lambda _{\text{ atom } }=0$)	1.2764	0.1512
Our method ($\lambda _{\text{ atom } }=0.5$)	0.8467	0.2402
Our method ($\lambda _{\text{ atom } }=1.0$)	0.9041	0.1886
Our method ($\lambda _{\text{ atom } }=2.0$)	0.8603	0.2279

**Table 4:** Experimental results in the unknown drug data sets from Kiba

Model	MSE	$R^{2}$
DeepDTA	0.5437	0.3605
GraphDTA	0.4950	0.2953
SAGDTA	0.6237	0.2311
MGraphDTA	0.4667	0.3766
Our method ($\lambda _{\text{ atom } }=0$)	0.7311	0.2039
Our method ($\lambda _{\text{ atom } }=0.5$)	0.4331	0.2831
Our method ($\lambda _{\text{ atom } }=1.0$)	0.4582	0.3906
Our method ($\lambda _{\text{ atom } }=2.0$)	0.6067	0.1781

**Fig. 4:**
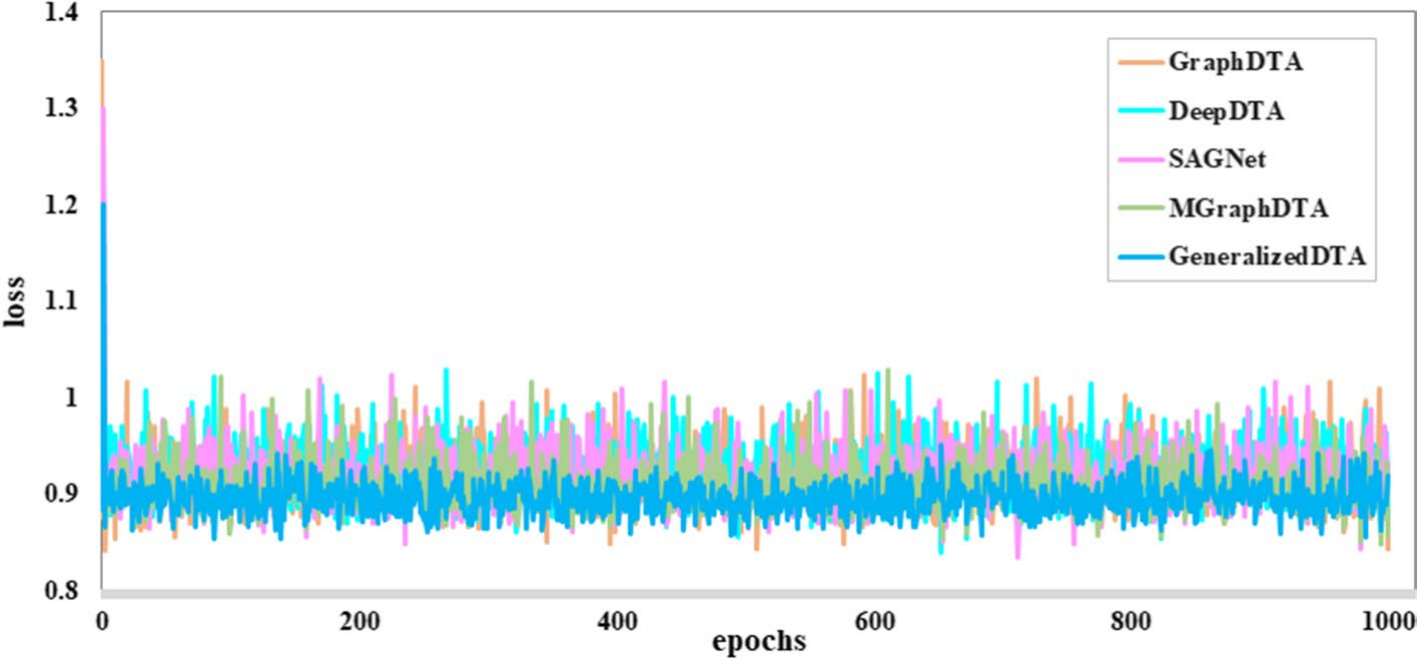
Convergence analysis in the unknown-TeS from Davis

Figure 4 gives convergence analysis in the unknown-TeS from Davis. As shown in this figure, the proposed GeneralizedDTA can effectively converge on the unknown-TeS from Davis and has the highest generalization capability.

## Discussion

### Ablation study

The proposed GeneralizedDTA combines pre-training and multi-task learning. It is involved with four core components, including protein pre-training, drug pre-training, multi-task framework and dual adaptation mechanism. In order to analyze their effectiveness, an ablation study is designed with 4 ablated variants, without protein pre-training, without drug pre-training, without multi-task framework and without dual adaptation mechanism. The variant without dual adaptation mechanism is to finish pre-training firstly, and then transfer the pre-trained components into DTA. Experiments were performed on the unknown drug data sets from Davis.

**Fig. 5:**
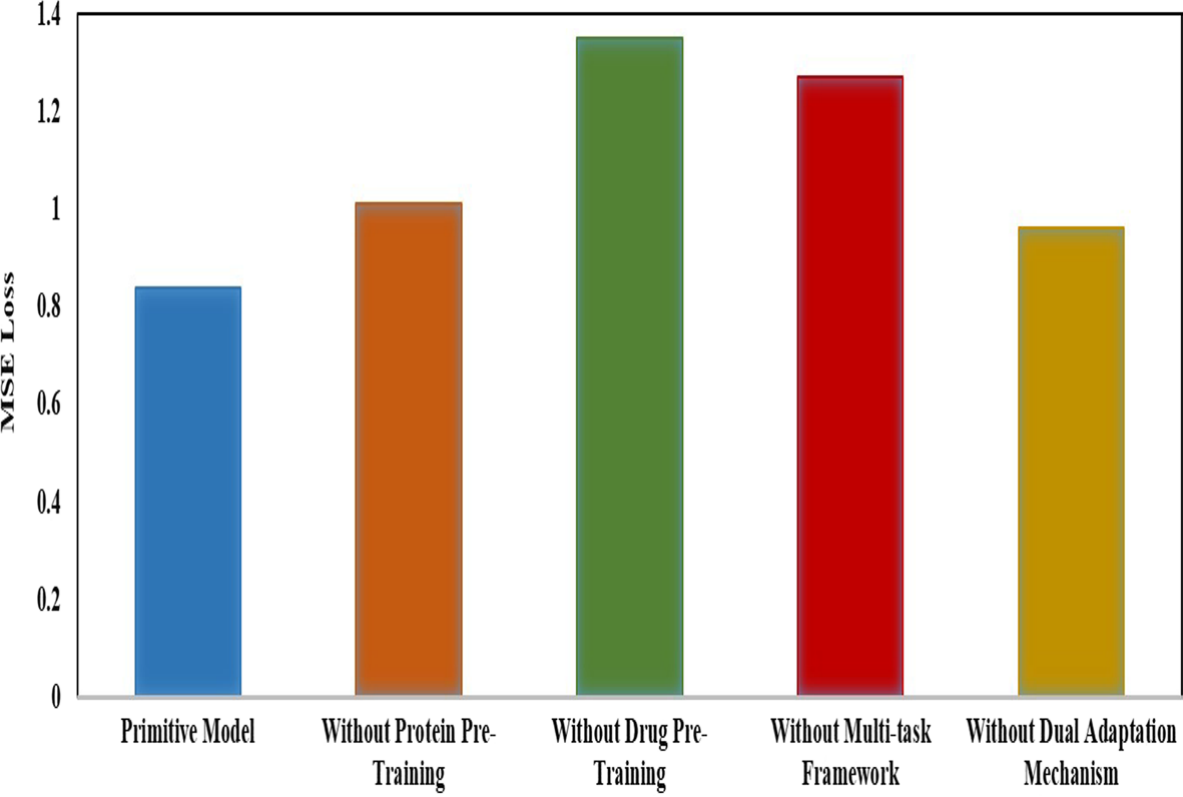
Experimental results in the ablation study

Figure 5 gives the experimental results. As shown in this figure, our method is superior to all variants. This indicates that all of four components are effective for improving DTA prediction. The effect of the drug pre-training is biggest and that of the protein pre-training is smaller. This indicates that the structural information of drug compounds is more important in DTA prediction than that of proteins. Figure 5 also shows that, the effect of the deep learning model can be significantly improved under the constraint of multi-task framework. Dual adaptation mechanism can prevent local optimality in parameter updating of deep learning model and improve performance of DTA prediction.

### Comparative analysis on pre-training models

Pre-training is a core component of GeneralizedDTA. This study adopted transformer-based protein pre-training and GCN-based drug pre-training. At present, there are several state-of-the-art protein pre-training models and drug pre-training models:

- ESM [46]: It is a protein pre-training model which uses a very large deep model framework with self-supervised task by masked language modeling and homology information relevant modeling.
- DISAE [47]: It is also a protein pre-training model which utilizes all protein sequences and their multiple sequence alignment to capture functional relationships between proteins without the knowledge of structure and function.
- ContextPred [48] : It is a drug pre-training model which explores distribution of graph structure in the node-level self-supervised task and sample subgraphs to predict their surrounding graph structures.
- GROVER [49]: It is also a drug pre-training model which uses local random walk-based objectives to learn rich structural and semantic information by self-supervised tasks in node, edge and graph level.

In order to evaluate pre-training of GeneralizedDTA, this study uses the above pre-training models to replace the pre-training components of GeneralizedDTA respectively. Experiments were performed on the unknown drug data sets from Davis.

**Fig. 6:**
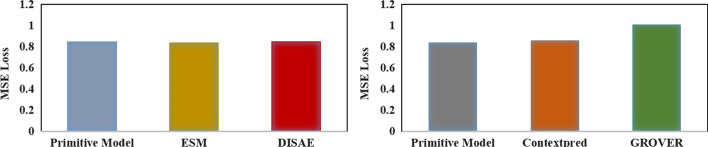
Comparative Analysis with different pre-training in unknown drug discovery

Figure 6 gives the experimental results. As shown in the figure, for protein pre-training, the DTA prediction result based on our transformer-based protein pre-training differs little from that based on ESM and DISAE. This indicates that these state-of-the-art protein pre-training models, such as ESM, can slightly improve DTA prediction, but not significantly. Considering the demand of computing resources, our protein pre-training is appropriate, especially in low resource environments.

Our GCN-based drug pre-training adopts the node-level self-supervised task. By randomly masked nodes and edge attribute [35], the GCN model can be trained to generate graph embedding which can distinguish the similarity of atoms. Based on this kind of graph embedding, the capability of downstream DTA prediction model can be effectively improved. As shown in Fig. 6, the DTA prediction results based on our GCN-based drug pre-training are similar to that based on ContextPred, but significantly better than GROVER. Because ContextPred also adopts the node-level self-supervised task, this indicates that node-level adaption surrounding neighbors in our GCN-based drug pre-training is more suitable for DTA prediction than the random walk strategy in GROVER. The reason may be that the random walk strategy pays too much attention to downstream irrelevant information.

The results of our model with $\lambda _{\text{ atom } }=0$ in Table 3 show that, if unbinding pre-training with prediction, the model can learn existing drugs too finely because of lacking of constraints, and lost the prediction capability on unknown drugs. Therefore, this study develops a multi-task learning framework with a dual adaptation mechanism to bind the drug pre-training and DTA prediction. In our dual adaptation mechanism, the parameters of GCN are not fixed every time. This helps to avoid falling into local optimization brought by the small labelled data set. We also use the loss function of the pre-training task as the regular term of the DTA prediction task to further alleviate overfitting of model. Therefore, the multi-task learning framework with a dual adaptation mechanism is most critical factor for improving the generalization capability of model on the DTA prediction of unknown drugs. The above comparative analysis on pre-training models also shows that only replacing the pre-training models cannot significantly improve DTA prediction. Considering the calculation complexity, the study adopts current transformer-based protein pre-training and GCN-based drug pre-training.

## Conclusion

Digging into the benchmark dataset Davis, we notice that previous studies on DTA prediction didn’t consider the generalization capability of model in unknown drug discovery. To address this challenge, this study proposes a new DTA prediction model called GeneralizedDTA. We introduce two protein pre-training tasks and a brand-new drug pre-training task to learn richer structural information of proteins and drugs, for accelerating the convergence of model on small-scale labelled data. We also develop a multi-task learning framework with a dual adaptation mechanism to prevent the prediction model from falling into overfitting and improve the generalization capability of model in unknown drug discovery. A group of comparative experiments on the new unknown drug data sets validate the effectiveness of our model for DTA prediction in unknown drug discovery.

## Data Availability

The source codes are publicly available in the GitHub repository https://github.com/Frank-39/GeneralizeDTA.
